# Optical modulation of nano-gap tunnelling junctions comprising self-assembled monolayers of hemicyanine dyes

**DOI:** 10.1038/ncomms11749

**Published:** 2016-06-08

**Authors:** Parisa Pourhossein, Ratheesh K. Vijayaraghavan, Stefan C. J. Meskers, Ryan C. Chiechi

**Affiliations:** 1 Stratingh Institute for Chemistry, and Zernike Institute for Advanced Materials, University of Groningen, Nijenborgh 4, Groningen 9747 AG, The Netherlands; 2 Molecular Materials and Nanosystems and Institute for Complex Molecular Systems, Eindhoven University of Technology, PO Box 513, Eindhoven 5600 MB, The Netherlands

## Abstract

Light-driven conductance switching in molecular tunnelling junctions that relies on photoisomerization is constrained by the limitations of kinetic traps and either by the sterics of rearranging atoms in a densely packed monolayer or the small absorbance of individual molecules. Here we demonstrate light-driven conductance gating; devices comprising monolayers of hemicyanine dyes trapped between two metallic nanowires exhibit higher conductance under irradiation than in the dark. The modulation of the tunnelling current occurs faster than the timescale of the measurement (∼1 min). We propose a mechanism in which a fraction of molecules enters an excited state that brings the conjugated portion of the monolayer into resonance with the electrodes. This mechanism is supported by calculations showing the delocalization of molecular orbitals near the Fermi energy in the excited and cationic states, but not the ground state and a reasonable change in conductance with respect to the effective barrier width.

## Abstract (web summary)

Flow of electricity between two electrodes can be tuned by monolayers of organic compounds. Here, Pourhossein *et al*. show that the tunnelling current of such molecular junctions can be modulated rapidly by illumination, using nanoskived gold wires separated by a self-assembled monolayer of hemicyanine dyes.

## Graphical abstract

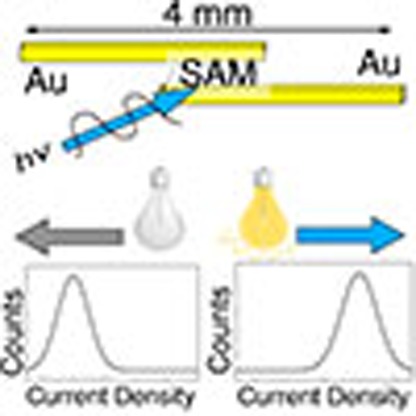

Devices in which the flow of electricity between two electrodes is modulated by organic compounds fall into two categories; bulk, thin-film devices and molecular, single-molecule. While both utilize synthetic chemistry to tune electrical properties, the latter combines molecular and electronic properties directly; rather than affecting charge-carriers en masse, tunnelling electrons directly utilize the orbital structure of individual molecules. Therefore, control over the composition, structure and conformation of molecules translates into device functionality. One of the most direct applications of this principle is photo-induced conductance switching, in which the tunnelling probability of a metal/molecule/metal junction is affected by photoisomerization *in situ*, typically leading to a change in tunnelling distance^1^. An immediate challenge to fabricating such devices is coupling light into the junction to affect the isomerization. The most common approach is to contact a self-assembled monolayer (SAM) with a (semi) transparent electrode such as graphene^2^,^3^, MoS_2_ (ref. 4), poly(3,4-ethylenedioxythiophene) polystyrene sulfonate^5^ or sufficiently thin Au (ref. 6). Care must be taken to choose a SAM that is sufficiently robust and that in which isomerization is not sterically inhibited^7^. An insurmountable problem with this approach to conductance switching is that the SAMs (and single-molecule junctions^8^) require long soak times to affect switching, usually on the order of tens of minutes. Symmetric dithiols can be self-assembled into enables on pre-fabricated gaps instead of planar electrodes, but the soak times increase^9^.

One approach to bring light-driven conductance switching into a useful time regime is to find systems that do not photoisomerize, but in which the tunnelling probability in the dark and light states differs. Such a system should exhibit photo-gating as opposed to reversible switching, the magnitude of tunnelling current should differ in the light and dark, and modulate on the timescale of a vertical excitation and relaxation. Battacharyya *et al*. measured the conductance of a fullerene-porphyrin dyad on indium tin oxide surfaces in break-junctions in the dark and under irradiation, observing a commensurate increase in tunnelling current that could not be attributed to photocurrent (the light-driven injection of carriers)^10^. They observed two distinct populations of conductivities, one low (dark) and one high (light). Under irradiation, the low-conductance population was depleted and the high-conductance population grew. This observation was only possible because of the long-lived state that results from the internal electron transfer from the porphyrin to the fullerene, allowing the coincidence of a dyad in the charge-separated excited state becoming trapped in the junction during an *I/V* sweep. The need for a transparent bottom electrode through which to irradiate the junction remains a challenge. In the aforementioned experiment, the authors used indium tin oxide, which is not suitable for forming high-quality SAMs for large-area measurements and devices. However, this result suggests that intramolecular charge-transfer may cause sufficiently significant electronic changes that can affect tunnelling charge transport in the excited state. And, while there are models that address the interaction of light with tunnelling junctions, theoretical development is hindered by experimental challenges^11^.

This paper describes the reversible, light-induced gating of tunnelling currents through nano-gaps between two parallel Au nanowires separated by a SAM comprising hemicyanine dyes. The monolayer serves both as the active component, affecting the change in tunnelling current, and as a template for the fabrication of the nano-gap. Hemicyanine dyes couple donor and acceptor units through a conjugated bridge, leading to strong optical absorption and a significant charge-transfer character^12^. We designed a series of hemicyanine dyes that form well-defined SAMs that are capable of producing photocurrent under illumination^13^. The origin of the photocurrent is the charge transfer that occurs on excitation, which is sufficient to drive electrons into the Au electrode that supports the SAM, while a redox couple collects the resulting hole. This series of compounds is, therefore, a good candidate for photo-gating and this class of compounds has even been investigated in tunnelling junctions (in the ground state)^14^,^15^. Rather than utilizing a transparent electrode, we incorporated these dyes into SAM-templated addressable nano-gap (STAN) electrodes^16^,^17^ in which a metal/SAM/metal sandwich structure is turned on its side and cut into thin sections by nanoskiving^18^,^19^,^20^. The resulting devices comprise two metallic nanowires in parallel that are separated by the cross-section of a SAM. The resulting structures are similar to the self-assembly of ensembles into pre-fabricated junctions^9^, but the process is bottom–up and can, therefore, accommodate asymmetric chromophores. (And STANs are millimetres in length.) Thus, the molecules spanning the electrodes are exposed and can be irradiated directly during *I/V* sweeps. On vertical excitation, molecules incorporating hemicyanine dye moieties enter a state of high conductance in which the density of states (DOS) of the frontier orbitals delocalizes, effectively decreasing the width of the tunnelling barrier without undergoing any bond rearrangements or conformational changes. Control measurements on SAMs of alkanes show no change in conductance.

## Results

### Effects of irradiation

**Figure 1:**
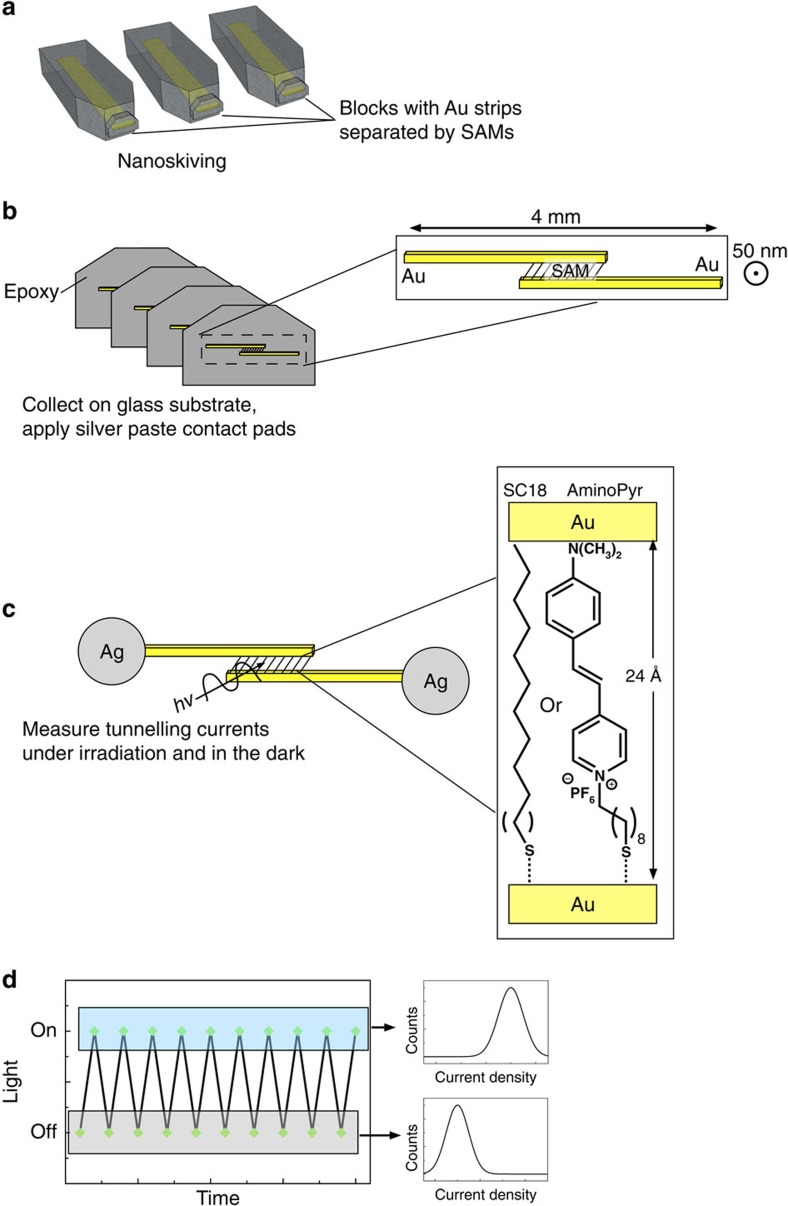
Schematic of the fabrication of STAN devices comprising SAMs of AminoPyr and C18. (**a**) Au/SAM/Au sandwiches are embedded in epoxy. (**b**) These epoxy blocks are sectioned by nanoskiving to form parallel nanowires separated by SAMs as depicted in the inset. (**c**) The sections are transferred to glass substrates and contact pads are painted onto the nanowires using silver paste to form the STAN devices comprising either SAMs of AminoPyr or C18 as depicted in the inset. (**d**) Tunnelling currents are measured for each device in the light and dark sequentially (dark-measure-light-measure...). Histograms of *J* for each value of *V* are constructed from the light and dark data. Details for the fabrication of STANs (steps **a**–**c**) are given in refs 16 and 17.

**Figure 2:**
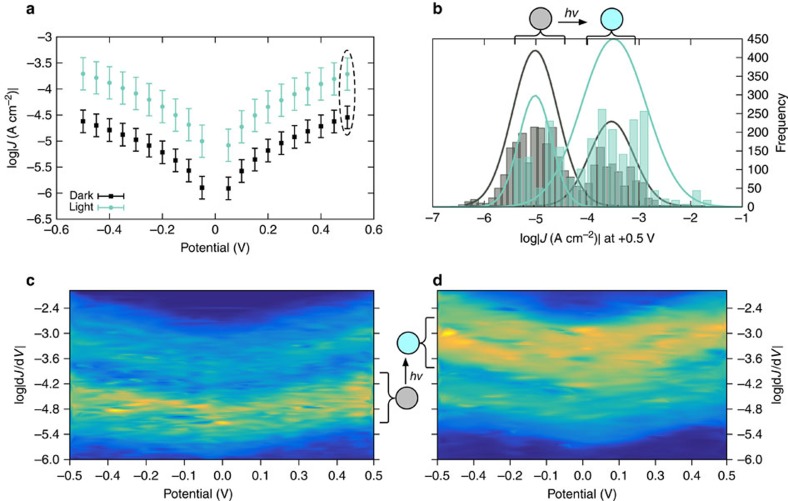
Summary of conductance data for AminoPyr in the dark and under irradiation. (**a**) Current-density versus voltage plots of AminoPyr-dark (back squares) and AminoPyr-light (cyan circles). The points correspond to Gaussian means from fits of histograms of log|*J*| for each value of *V*. The error bars are the 95% confidence intervals (see Supplementary Methods for details). (**b**) The histograms and Gaussian fits of log|*J*| at +0.5 V (corresponding to the data in the dashed oval in the top-left plot). (**c**) Conductance heatmap plot for AminoPyr-dark showing histograms binned to log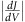 (conductance, *y* axis) versus potential (in V, *x* axis). The colours correspond to the frequencies of the histograms; lighter colours indicate higher frequencies (yellow ∼450.) (**d**) An identical heatmap plot for AminoPyr-light. The coloured circles correspond the the data in the dark (grey) and under illumination (cyan). A schematic of the junction is shown above the top-left plot and the structure of AminoPyr is shown between the top two plots. These data show a clear, statistical difference between AminoPyr-dark and AminoPyr-light.

The device architecture and experimental design are shown in Fig. 1. The tunnelling current of STAN electrodes incorporating SAMs of the hemicyanine dye 1-(10-acetylsulfanyldecyl)-4-[2-(4-dimethylamino-phenyl)vinyl] quinolinium hexfluorophosphate (AminoPyr, shown in Fig. 1) increases during exposure to light. The current of STANs incorporating octadecanethiol (C18) is insensitive to light. We measured the photo-gating effect using lasers of three different wavelengths, 405, 532 and 650 nm. Due to the broad absorption of hemicyanines and the further broadening that occurs on adssoprtion to Au, all three wavelengths fall within the absorption spectrum of AminoPyr (Supplementary Fig. 1). We observed no significant difference between them and, therefore, present the light data as an aggregate data set of three lasers. (An important distinction between gating and switching is that switching requires two wavelengths to affect the two directions of switching; gating results in a high-conductance light state and a low-conductance dark state.) From these raw *J*/*V* data, we constructed histograms of log|*J*| for each value of *V* with the laser light switched on and off (which we refer to as ‘light' and ‘dark' respectively). The peaks of these histograms (*μ*_log_) and the 95% confidence intervals (computed from *σ*_log_) are shown in the top left of Fig. 2. The histograms of all *J*/*V* data are shown in Supplementary Fig. 2. Every measurement under illumination did not lead to an increase in conductance nor did every measurement in the dark produce the same conductance. Rather, two populations emerged for AminoPyr, one centred at ∼10^−5^ and one at ∼10^−3^ A cm^−2^ for measurements in the light and dark, respectively. The population in the dark is approximately inverted in the light; under illumination the population at 10^−5^ depletes and the one at 10^−3^ grows. This inversion is visible in the bottom of Fig. 2, which shows conductance heat maps formed from histograms of log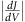 versus *V* in which the colours correspond to the frequencies of the bins, from dark blue (0) to yellow (∼450). These plots show, in one picture, the entire data set; each point of the plots corresponds to a conductance (*G* as log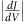) at a particular voltage and the colour indicates the frequency with which that *G*/*V* pair was observed. In the case of AminoPyr, the heatmap plots in Fig. 2 show two clear tranches of conductance with different frequencies that captures the entirety of the photo-gating phenomenon. The top right of Fig. 2 shows histograms and Gaussian fits of *J* at +0.5 V for AminoPyr in the light and dark showing the population inversion more clearly. The fits were made by selecting the peaks of the histograms, that is, with the assumption that the histograms in the light and dark each describe a bi-modal distribution.

**Figure 3:**
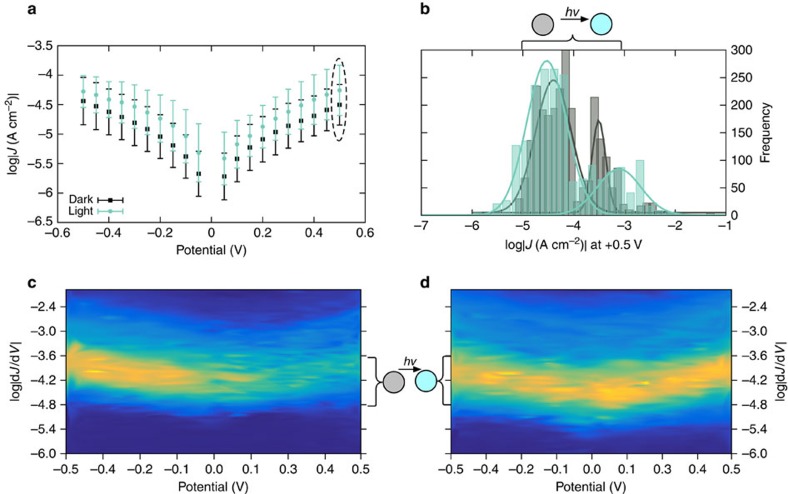
Summary of conductance data for C18 in the dark and under irradiation. Plots of C18 constructed identically to those shown for AminoPyr in Fig. 2. (**a**) *J*/*V* curves in the light and dark. (**b**) histograms of *J* at +0.5 V in the light and dark. Conductance heatmap plots in the dark (**c**,**d**). These plots show that there is no statistical difference between C18-dark and C18-light.

Figure 3 shows conductance heatmap plots of STANs comprising C18 (bottom) with a population centred at ∼10^−4.2^ and a tail extending to ∼10^−2^ A cm^−2^ in the dark, however, the population does not change under illumination. The top right plot shows Gaussian fits made with the assumption that the histograms in the light and dark each describe a bi-modal distribution for the sake of comparison to AminoPyr; the conductance heat maps of C18 clearly show only one tranche for each state. Unlike AminoPyr, the magnitude of the two Gaussian fits does not change; fits around −4.5 are almost identical in the light and dark. From the plots in Figs 2 and 3 we can draw three important conclusions: first, the magnitude of the conductance in the dark is roughly length-dependent because both C18 and AminoPyr are approximately the same length and exhibit the same conductance (in the dark); Second, only STANs comprising AminoPyr show two distinct tranches of conductance and the populations of these tranches approximately inverts between the dark and light states; Finally, all of the STAN devices investigated in the light and the dark exhibit approximately symmetric, bowl-shaped conductance plots with positive curvature, which indicates that the dominant mechanism of conduction is likely tunnelling (which we previously established for STANs of alkanes^16^). Although the curvature of the plots of AminoPyr and C18 is slightly different, the shape of AminoPyr-dark and AminoPyr-light remains unchanged, which is a strong indication that the absorption of light increases the tunnelling probability and is not the result of photocurrent or the appearance of a hopping mechanism.

### Conductance gating

While there are no established methodologies for characterizing light-induced changes in conductance in tunnelling junctions, a figure of merit in any device configuration is reversibility. Typically *J* at some voltage is plotted against either the number of cycles (or time at a constant rate of switching). Since virtually all conductance switching is based on (photo)isomerization, reversibility is generally limited by fatigue of the SAM; irreversible pathways, disorder, degradation and so on. Typical devices last for fewer than 10 cycles^2^,^4^,^5^,^6^, with the notable exception of a graphene/monolayer/graphene system based on azobenene isomerization that switches for at least 50 cycles^3^. Direct comparisons with our photo-gating measurements are difficult because of the statistical nature of the observation of switching and the completely different mechanism by which the change in conductance occurs. Battacharyya *et al*. were only able to report conductance histograms in the light and dark (which are sufficient to demonstrate the switching phenomenon) because of the nature of break-junction measurements^10^. Since each observation is sampling the junction in one, specific state (a particular binding mode or the absence of a molecule), the peak conductance—and the gating effect—can only be discerned by assembling a histogram of events (plateaux of *G*) after many thousands of observations. Our STAN junctions, however, are monolithic devices and conductance switching occurs in-place, thus we report the statistics of switching events rather than the formation of junctions.

**Figure 4:**
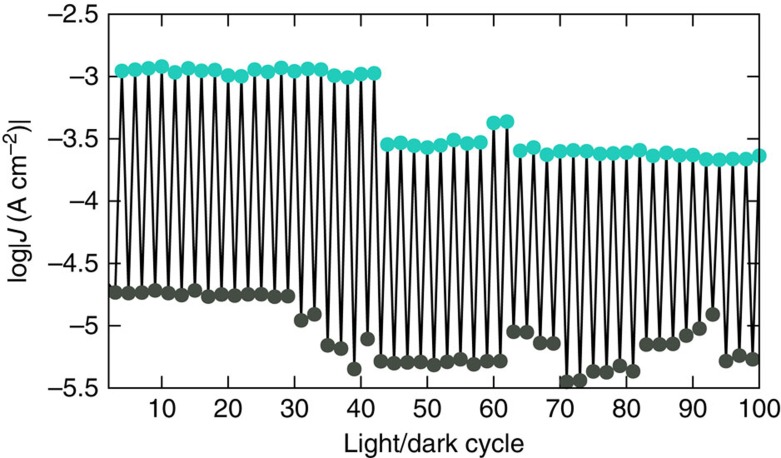
Plot of conductance cycling of AminoPyr. The values of log|*J*| at +0.5 V as a function of light/dark cycles for a range of 100 consecutive cycles. The cyan circles correspond to log|*J*| under irradiation and the black circles to log|*J*| in the dark. The time between each cycle is on the order of 60 s. The entire range (1,000 cycles) is shown in Supplementary Fig. 3.

We acquire data sequentially and build up conductance histograms by collecting many values of *J* at each value of *V* for a device in a particular state that is constant with the exception of the changing variable of illumination. That is, we can construct a plot of *J* in time similar to those reported for conductance switching based on photoisomerization. However, we do not always observe switching either because it did not occur (due to a subtle change with the laser, a ‘bad' SAM, or some other uncontrollable variable) or because that particular device was under-sampled when it failed; as Fig. 2 shows, the histograms in the light and dark overlap slightly. Thus, what we show in Fig. 4 is 100 consecutive cycles in which switching was observed for each ‘on/off' event. Sequences of 100 cycles can span at most two devices (because each was cycled ∼100 times), but the average time between cycles is ∼60 s, which is limited by the integration time of the *J*/*V* sweeps. The full range of 1,000 and a plot showing that the apparent gating of C18 was smaller in magnitude and not in phase with irradiation are shown in the Supplementary Figs 3 and 4 and discussed in Supplementary Note 1. (The statistical proof of photo-gating is captured in Fig. 2.) The failure mode of our devices is the collapse of the STAN electrodes due to electrostatic stress (and/or the migration of Au atoms) because there is no fatigue associated with exciting a dye molecule on the order of 100 times. Isomerization causes fatigue of the SAM, which typically leads to dampening of the *J*/*t* plots, while our devices work for a finite number of *J*/*V* cycles irrespective of switching events and then short abruptly. The yields of working STAN devices are not straightforward to quantify because they oscillate between 100 and 0% as the knife cuts through a defect in the SAM. Typically, when we encounter a shorted device, cleaving ∼1 μm from the block results in non-shorted devices, but the duration of spans of working devices depends on the identity of the SAM. Typical average yields for long-chain SAMs are ∼70% (ref. 16).

**Figure 5:**
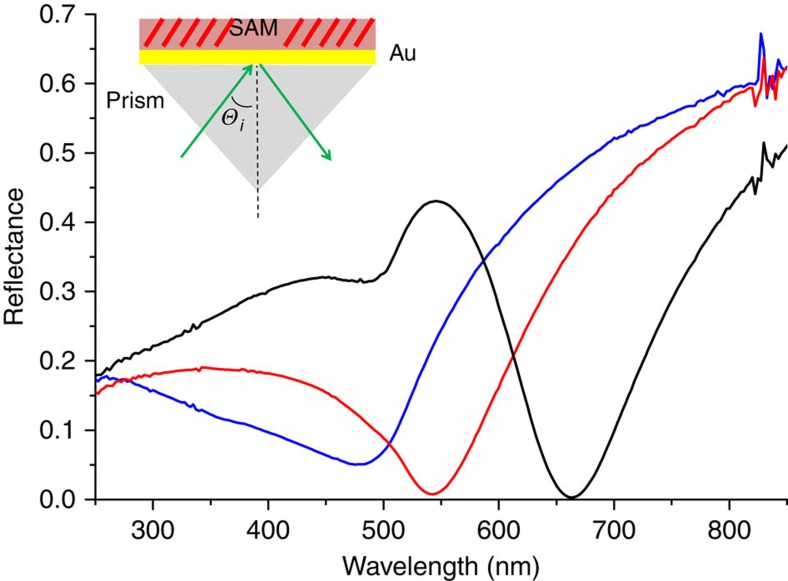
Angular dependence of reflectance for SAMs of AminoPyr on Au. Decreases in reflectance indicate Plasmon absorption, showing that Plasmon absorptions occur across all the wavelengths used to excite the STAN devices. The different curves correspond to different values of Θ_*i*_: 80° (blue), 55° (red) and 47° (black). The absorbance becomes more pronounced and red-shifted at 47°, which is comparable to the angles used to irradiate the STAN devices.

The microscopic mechanism of the photo-gating phenomenon is not possible to derive from conductance data alone, but we can reasonably exclude some possibilities. We were unsuccessful in resolving *J* in time with a lock-in amplifier and thus can only state that the experimentally observed switching time is <60 s (the time resolution of our measurement). And the only other data point we were able to obtain from the series of hemicyanines (OMePyr, Supplementary Note 2) did not provide statistically significant differences in *J* (Supplementary Figs 5–7). The lack of wavelength dependence is not surprising if one considers the cross-section of the SAM compared with the electrodes; ∼1 nm of SAM compared with 100 nm for each electrode. Thus, the absorption of light may take place at the electrodes, which transfer energy to the SAM. Surface-enhanced Raman spectroscopy of molecules adsorbed to Au STAN electrodes shows a strong dependance on the width of the gap with a maximum at 5 nm (ref. 21). Figure 5 shows the angular dependence of reflectance of SAMs of AminoPyr on planar Au. There is a strong dip centred between ∼450 and ∼650 nm corresponding to Plasmon absorption that shifts to lower energies at 47°, which is within the range used to irradiate the STAN electrodes (the surfaces of the two electrodes parallel to the substrate). At 47° the normalized reflectance begins to drop rapidly at ∼700 nm, reaching a minimum at 650, mirroring the absorbance spectrum shown in Supplementary Fig. 1. Given that photo-gating requires only the absorption of a photon to induce a high-conductance (excited) state, it is reasonable to expect that irradiation of a Au substrate support a SAM of AminoPyr will lead to a fraction of molecules populating this state. In the absence of any dramatic shifts in absorbance induced by the Van der Waals contact to the ‘top' electrode in the assembled STAN devices irradiation at wavelengths below ∼700 nm would, therefore, induce a high-conductance state by energy-transfer from the Plasmon to the SAM. And Plasmon energy-transfer processes have been observed for SAMs of AminoPyr in a liquid-cell photovoltaic device^22^.

Finite-element analysis of STANs shows a significant field enhancement in the gap as compared with the edges, suggesting that, in addition to the Plasmon modes present in planar substrates, STANs can act like antennas, directing energy into the gap containing the SAMs (ref. 23). This field enhancement alone could modulate the conductivity of the gap; tunnelling current can be generated directly from plasmon resonances across metallic nano-gaps bridged by dithiols at 0.6−1.0 eV in the terahertz regime^24^. That mechanism is unlikely in our case given the difference in energies and the fact that it would be present in C18, for which we observe no increase in *J* on irradiation. Likewise any thermal effects arising from the irradiation of the STAN electrodes would have to arise from the SAM of AminoPyr. Although we cannot rule out the thermal excitation of AminoPyr, it would still constitute a photo-gating phenomenon; only the mechanism of energy-transfer from the light would differ.

The lack of any obvious wavelength dependence could be due to the broad absorption of the Plasmon mode; given the proximity of the molecules to the electrode (the *π*–system is in direct contact), sufficient near-field modes would be active to conceal a weak-wavelength dependance from the direct absorption of chromophores. However, a difference in absorption would correspond to a difference in the fraction of molecules in the light state and *J* saturates very quickly as a function of the fraction of excited chromophores (Supplementary Fig. 8 and Supplementary Note 3). Changes in log|*J*| of less than *σ*_log_, if present, would require testing hundreds of devices to resolve statistically. Thus, the expected behaviour irrespective of the details of light absorption and energy-transfer is a threshold whereby *J* jumps from a low-conductance to a high-conductance state at a critical intensity of light independent of wavelength below ∼700 nm. Such behaviour is fully consistent with our observations.

**Figure 6:**
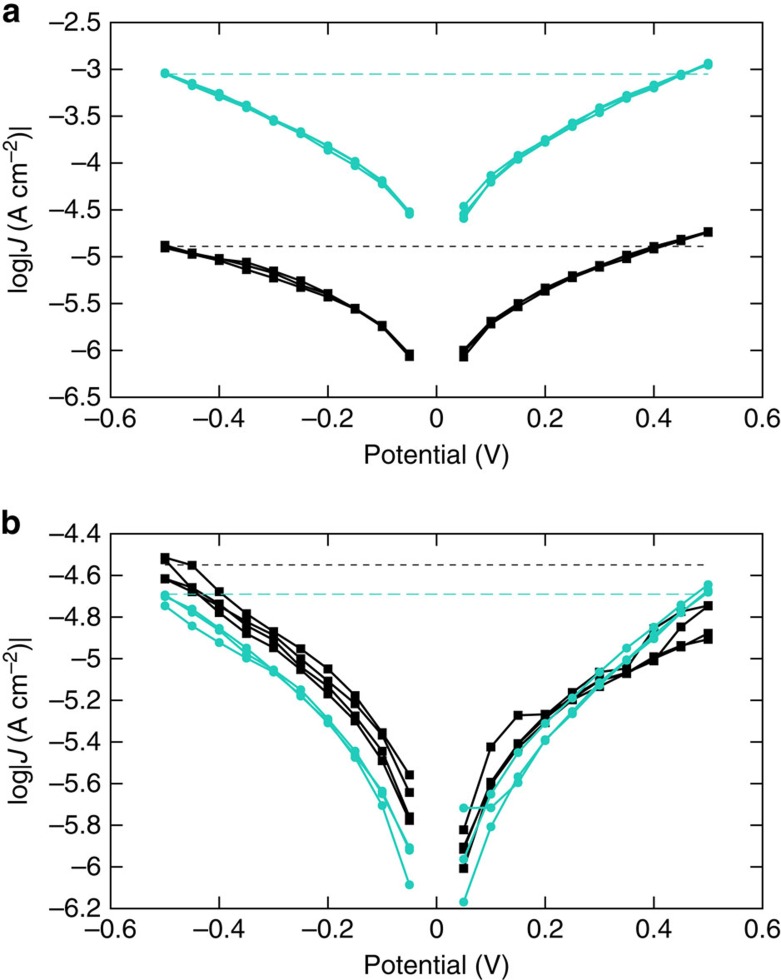
Individual *J*/*V* plots of AminoPyr and C18. Plots of log|*J*| versus *V* for AminoPyr (**a**) and C18 (**b**) from a single device showing three traces each from a single dark–light–dark–light–dark–light cycle. The dashed lines show the slight asymmetry of the device in both the dark (black squares) and light (cyan circles) states.

Another possibility is that, regardless of the mechanism, once promoted to an excited state, the chromophores simply inject an electron (or hole) into the adjacent electrode, giving rise to an increase in current. If this current were of sufficient magnitude to observe as an increase in *J*, it would almost certainly show a difference in the symmetry of the *J*/*V* curves in the light and dark states and/or a measurable photo-voltage; neither of these are present. Figure 6 shows no difference in the *J*/*V* curves of AminoPyr-dark and AminoPyr-light, and very little difference in symmetry (ratio of log|*J*| at ±0.5 V) between AminoPyr and C18. In fact, C18 is slightly more asymmetric and in the opposite direction as AminoPyr. (The asymmetry in both cases probably arises from the disparate interfaces.) We propose instead that the change in conductance is due to the excited-state singly occupied molecular orbital (SOMO) of AminoPyr-light coming into resonance with the electrodes and reducing the effective tunnelling distance to that of the alkyl tail as depicted in Supplementary Fig. 9.

A simple way to model a metal/SAM/metal tunnelling junction in which the SAM contains molecules in different conductive states is to treat these molecules as ‘thin-area' defects^25^ or, in our case as ‘high-*J* defects.' In the dark, the expectation is that the entire population of molecules is in a nominally low-conductance state defined by the thickness of the SAM and the details of the tunnelling barrier. When a fraction of molecules populates a high-conductance state, they can be considered high-*J* defects because, although they do not change in length, the width of the tunnelling barrier decreases and they exhibit an increase in conductance analogous to the decrease in distance that occurs in, for example, photo-induced *cis*/*trans* isomerization of an azo-benzene. Thus, in the light, the expectation is that a significant fraction of the population is in a high-*J* state and carries most of the tunnelling current. This model predicts that high-*J* defects have a pronounced influence on the total magnitude of *J* that scales with the decrease in effective tunnelling distance (but not light intensity), while the remainder of the SAM—in the ground state—remains in the low-*J* state. According to this model, if the conductance of AminoPyr-light (high-*J*) corresponds to an effective distance of 55% of AminoPyr-dark (low-*J*), only 2% of the population of molecules in the SAM need be in the AminoPyr-light state for them to carry >85% of the total current through the junction. Thus, a steady-state of population consisting of 98% dark-state and 2% light-state (excited or charge-separated) molecules of AminoPyr could cause the higher value of *J* that we attribute to AminoPyr-light. The threshold population necessary to affect a measurable difference in *J* varies inversely with the difference in conductivity between the dark and light states (the change in effective distance), but it only reaches 10% at a difference in conductivity that corresponds to an effective distance of 50%. To put this estimate into context, an azo-benzene switch (based on photoisomerization) showed a change in *J* of 10^1.4^ A cm^−2^ with a change in distance of 35% (ref. 6) and a change in *J* of 10^1^ A cm^−2^ with a change in distance of 21% (ref. 3). We observe a maximum difference in *J* of ∼10^2^ A cm^−2^ for AminoPyr, for which the chromophore comprises ∼50% of the total molecular length (these data are plotted in the Supplementary Figs 8 and 10). This model is discussed in detail in Supplementary Note 3; using experimentally derived parameters, it correctly predicts the observed change in *J* via the mechanism described above.

**Figure 7:**
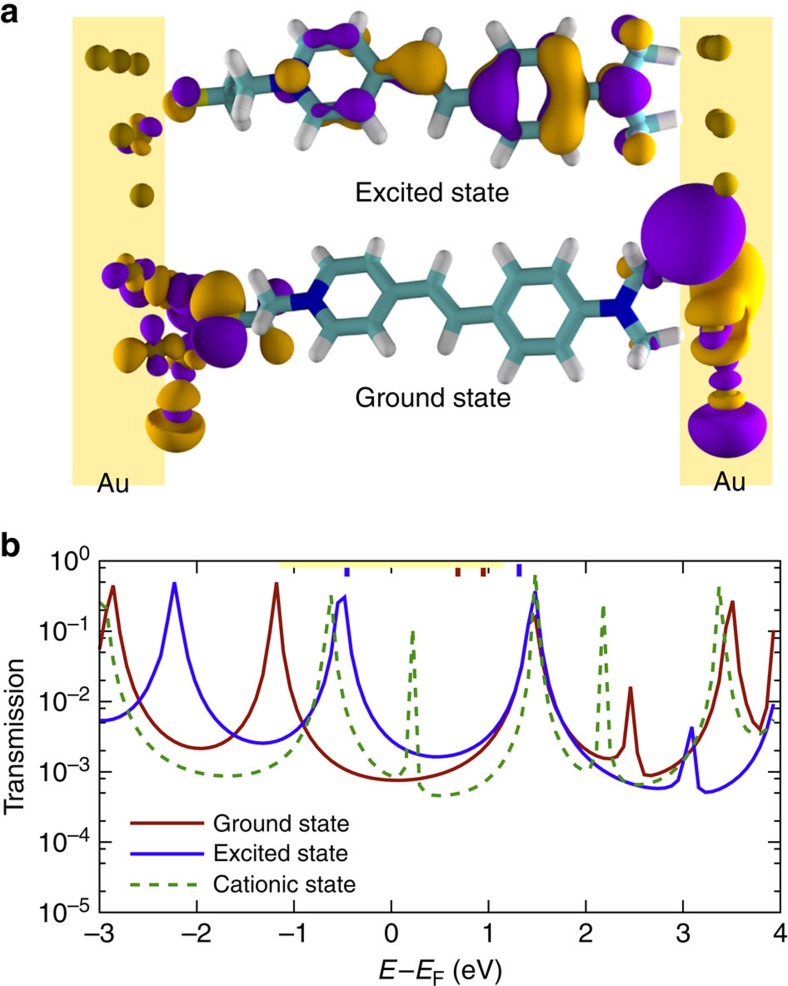
Calculated molecular orbitals and transmission of AminoPyr between Au electrodes. (**a**) The frontier orbitals of AminoPyr with truncated alkyl chains between two six-atom Au clusters in the ground and photo-excited states (Supplementary Note 2). The frontier orbitals of the cationic state are identical to the excited state. (**b**): The transmission spectra of the same structures in the ground, excited and cationic states with *E*_F_ set to −5.0. The positions of the frontier orbitals for the calculated structures are indicated with thick lines across the top of the plot. The yellow bar spans a 2 eV window centered at 0. Because the frontier orbitals only span the electrodes in the excited and cationic states, exciting AminoPyr or formally transferring and electron to the electrode shift a highly transmissive feature from outside to inside the bias window in agreement with experimental observations. In the cationic state an additional feature appears very near *E*_F_.

The high-*J* defect model explains how photo-excitation can lead to an overall increase in *J*, but it is silent on the underlying mechanism by which a molecule in the excited state is more conductive in a tunnelling junction. To elucidate that mechanism, we performed density functional theory (DFT) calculations on AminoPyr in the ground and (triplet) excited state as well as the formally charged state in which an electron is transferred to the electrode on excitation as has been observed for SAMs of AminoPyr in dye-sensitized photovoltaic devices^13^. For computational simplicity (as discussed in Supplementary Note 4) we simulated the electrodes as six-atom Au clusters and truncated the alkane spacers to two carbons, which is sufficient to decouple the thiol-electrode interface from the hemicyanine-electrode interface. These data are summarized in the top panel of Fig. 7 and show that the density of states (DOS) derived from the frontier orbitals is completely localized to the electrodes and the alkane spacer in the ground state in agreement with the model proposed above; tunnelling is off-resonant and dominated by molecular length in the ground state. In the excited state, this DOS delocalizes across the entire molecule, effectively hybridizing the hemicyanine chromophore with the electrode. The DOS of the cation (not pictured) is identical. These observations further support the proposed model; in the excited state, the hemicyanine (55% of the total tunnelling distance) presents a significantly lower or absent tunnelling barrier as its frontier orbitals are engaged. The bottom panel of Fig. 7 are simulated transmission curves (with *E*_F_ set to −5.0 eV) in the ground, excited and cation states. These data show two main resonance peaks, both of which are outside of or on the edge of the bias window in the ground state. In the excited state, the feature below *E*_F_ shifts almost 1 eV towards the centre of the bias window (*E*_F_). This shift is also visible in the cation state together with an additional resonance just above *E*_F_. Thus, these transmission curves support the hypothesis that AminoPyr-light is more conductive than AminoPyr-dark whether or not AminoPyr becomes oxidized.

## Discussion

We observed the photo-gating of molecular tunnelling junctions comprising hemicyanine dyes in a STAN electrode configuration. These are monolithic devices that can be fabricated on-demand and the entire life cycle from fabrication to wiring to measurement occurs under ambient conditions; no clean rooms, high vacuum set-ups and so on. (except for the initial deposition of Au). This configuration also obviates the need for a transparent electrode by exposing the ‘side' of a SAM between two parallel nanowires. While there are some top–down techniques that similarly expose the sides of molecules^26^, they are limited to symmetric molecules, are not compatible with SAMs and are gated with electric fields instead of light. Thus, STAN electrodes are unique in their ability to allow the interrogation of tunnelling junctions comprising SAMs (and asymmetric molecules) with light. Control experiments on junctions incorporating alkanedithiols show no evidence of photo-gating, thus we can rule out the electrodes as the source of the change in conductance. We can therefore ascribe the photo-gating effect to the SAM and propose a mechanism whereby the fraction of molecules in the light-state modulate the conductance of the entire junction. This model correctly predicts the observed magnitude of *J* in the light and agrees with literature values of changes in *J* on photoisomerization.

While control experiments on SAMS of C18 rule out effects arising from the electrodes, the lack of the ability to characterize the SAMs in an assembled STAN device hinders proving the proposed mechanism directly. Plasmons probably play a role, as modes are active across the entire absorption window of SAMs of AminoPyr and surface-enhanced Raman effects have been observed in STAN electrodes. The lack of an obvious wavelength dependence supports the hypothesis that Plasmons are involved in absorption and energy-transfer processes, but further experiments on sets of molecules in which the identity and dimensions of the electrodes are varied are needed to elucidate the mechanism fully. The lack of an obvious dependence of the magnitude of the change in conductance on the intensity of excitation (and/or absorbance as a function of wavelength) is consistent with the proposed mechanism of high- and low-J ‘defects' modulating the conductance of the entire junction, which is in turn supported by DFT calculations. Properly testing this hypothesis will, however, require synthesizing series of dyes in which the relative lengths of the chromophore and alkyl spacer are varied. Relating the exact mechanism of light absorption and energy-transfer to the magnitude of conductance switching is a matter for future investigation, as this paper only presents robust experimental evidence of a photo-gating phenomenon in solid-state, bottom devices fabricated by nanoskiving.

The proposed photo-gating mechanism does not involve isomerization or the rearrangement of atoms, thus the gating time is much shorter than ordinary switching and does not show rapid fatigue. It therefore differs from previous reports of conductance switching for which the mechanism is generally attributed to a change in effective tunnelling distance following a *cis*/*trans* isomerization or a change in conjugation patterns with ring opening/closure. Thus, while switching requires several minutes of soak time to affect a chance in conductance, photo-gating is limited only by the timescale of a vertical excitation and relaxation back to the ground state. Simulated transmission spectra and calculated DOS derived from the frontier orbtials show that tunnelling probability (conductivity) of AminoPyr is higher in both the excited and cationic states. A vertical excitation and/or quenching by charge-transfer to the electrode can, therefore, increase the conductivity and we can reasonably assert that the change in conductivity occurs on the order of picoseconds. This work represents a step towards realizing functional devices based on molecular tunnelling junctions, in this case devices that can respond to light in real-time.

## Methods

### STAN electrodes

The nano-gap devices were fabricated by sectioning sandwich structures of Au/SAM/Au where SAM is a SAM of either C18 or AminoPyr using an ultramicrotome (nanoskiving). A representative scanning electron micrograph is shown in Supplementary Fig. 11. The SAM was grown on the buried interface of a thin film of Au that was template-stripped from a technical grade Si wafer. The second Au layer was deposited by thermal vapour deposition directly onto the Au/SAM structure. The fabrication and electrical measurement of STAN electrodes incorporating C18 is also described in detail elsewhere^16^,^17^. A representative s.e.m. image of a STAN of AminoPyr is shown in Supplementary Fig. 10 The limiting step in the fabrication is the deposition of Au directly onto the SAM that serves a template for the gap. We were able to fabricate STANs of AminoPyr and OMePyr successfully, but SAMs of the other hemicyanine compounds in the series^13^ proved too fragile to serve as gap templates. We collected the STAN electrodes on 1 cm glass substrates in ‘ribbons' of 5–10 devices from the water bath of the diamond knife that we used for nanoskiving. After drying the ribbons at 60 °C for 1 h, we painted silver paste contacts onto the ends of the wires (as depicted in Fig. 1), which we contacted inside a home-built Faraday cage equipped with an electrometer and a moveable stage. We investigated the STAN devices by sweeping the potential between ±0.5 V and collecting *J*/*V* curves while alternating the light conditions between dark and irradiation with a diode laser. After a sufficient number of *J*/*V* sweeps or when a device failed, we continued the measurement on the next device by advancing the stage and contacting the next device (large data sets across multiple devices were acquired in rapid succession). A more detailed description of the experimental procedure is provided in the Supplementary Note 2.

### *J*/*V* measurements

We collected *J*/*V* data in four different situations: (1) irradiation with green light (*λ*=532 nm), (2) irradiation with purple light (*λ*=405 nm), (3) irradiation with red light (*λ*=650 nm), (4) no irradiation (dark). All lasers are continuous wave (CW), <1 mW. The angle at which the light struck the substrates on which the devices were mounted varied between 30 and 60°. In static photo-switching studies each device is interrogated first in a stable ground state and then, after several minutes of irradiation, interrogated again in a (kinetically trapped) switched state. Since our devices do not rely on photoisomerization, we characterized their *J*/*V* properties by interrogating them sequentially in the dark and under illumination. That is, for each device we acquired several *J*/*V* traces under ambient conditions, switched on the laser and acquired several more, switched if off and measured several more and so on, until we reached ∼100 switching events or the device shorted. The magnitude of the currents were in the pA-nA regime and required both amplification and integration, which takes place in a Keithley 6430 electrometer. As a result, we were unable to resolve the current in time with greater resolution than one *J*/*V* sweep, which is on the order of one minute.

### Data availability

All of the data used to prepare this manuscript and the Supplementary Information are available upon request.

## Additional information

**How to cite this article:** Pourhossein, P. *et al*. Optical modulation of nano-gap tunnelling junctions comprising self-assembled monolayers of hemicyanine dyes. *Nat. Commun.* 7:11749 doi: 10.1038/ncomms11749 (2016).

## Supplementary Material

Supplementary Information Supplementary Figures 1-11, Supplementary Notes 1-4, Supplementary Methods and Supplementary References
