# Supplementary material for: Optical modulation of nano-gap tunnelling junctions comprising self-assembled monolayers of hemicyanine dyes
Source: Nat Commun. 2016 Jun 8;7:11749. doi: 10.1038/ncomms11749 (PMC4899853; doi:10.1038/ncomms11749)
Supplement: Supplementary Information — Supplementary Figures 1-11, Supplementary Notes 1-4, Supplementary Methods and Supplementary References [file ncomms11749-s1.pdf]

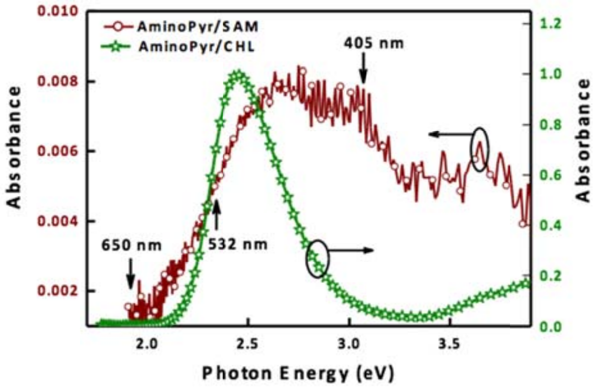

Supplementary Figure 1: UV-Vis absorption of AminoPyr in ($CHCl_3$) and in a SAM on Au. The indicated wavelengths correspond to the lasers used for excitation in the photo-gating study. Although 405 and 650 nm both lie outside of the absorption spectrum in solution, the broadening that occurs upon binding to Au places them both inside the absorption window.

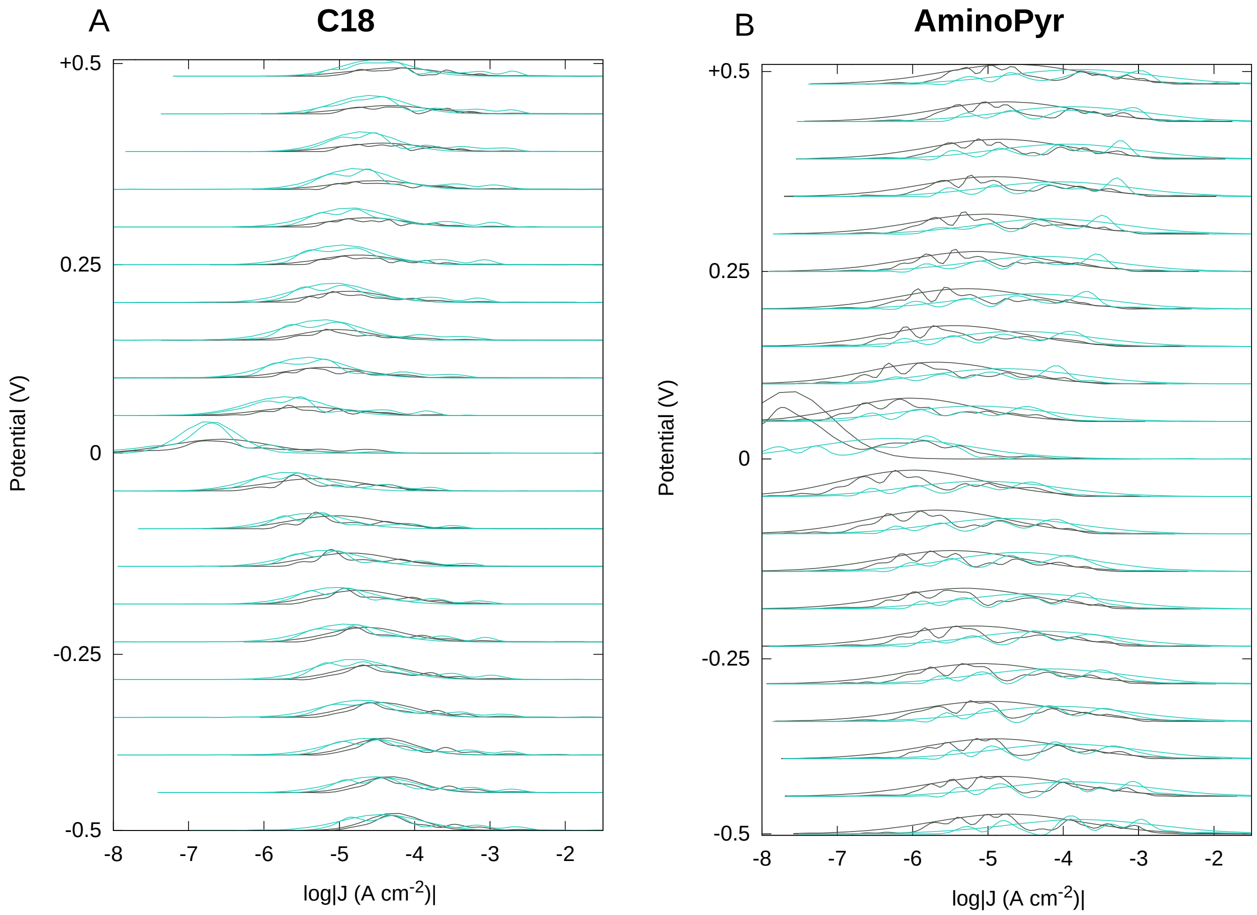

Supplementary Figure 2: Voltage–current characteristics of AminoPyr-dark and C18 nanojunctions. Pseudo-3D plots of the raw data showing histograms (solid lines) and Gaussian fits (filled curves) of all of the J/V data for AminoPyr-dark and C18-dark (black) and C18-light and AminoPyr-Light (cyan). The pseudo-Y axis corresponds to the potential for each histogram and the X-axis are the bins in units of log|J (A/cm^{-2})|, *i.e.*,the plot resembles a *J/V* rotated by 90 °. The histograms and fits for C18-dark and C18-light are completely overlapping (left plot), while the

histograms and fits for AminoPyr-light are shifted to higher values of log|*J*| relative to AminoPyr-dark (right plot).

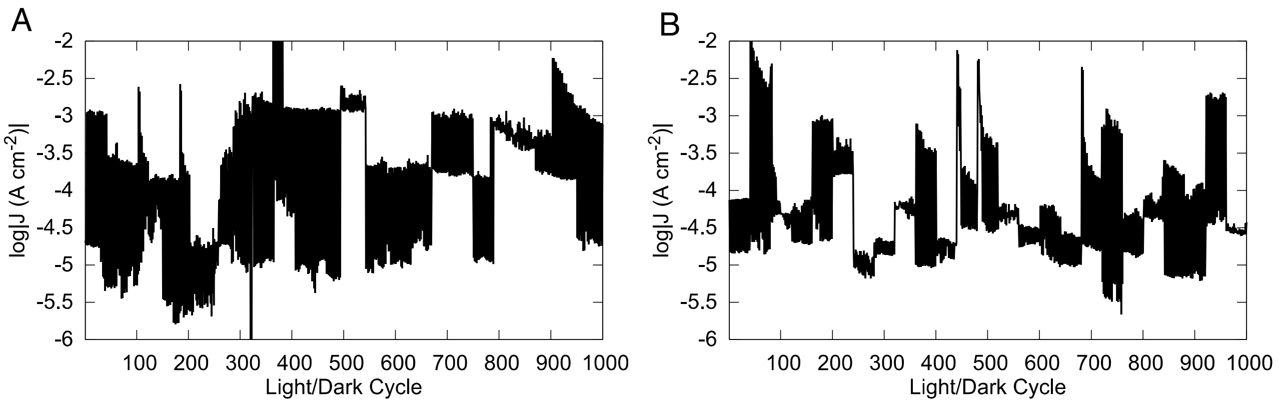

Supplementary Figure 3: Influence of illumination on current density through AminoPyr and C18 nanojunctions. The values of log|*J*| at +0.5 V as a function of light/dark cycles for the entire dataset for A: AminoPyr and B: C18. Qualitatively there is a clear difference in the two plots; AminoPyr shows significantly more events than C18, see Supplementary note 4.

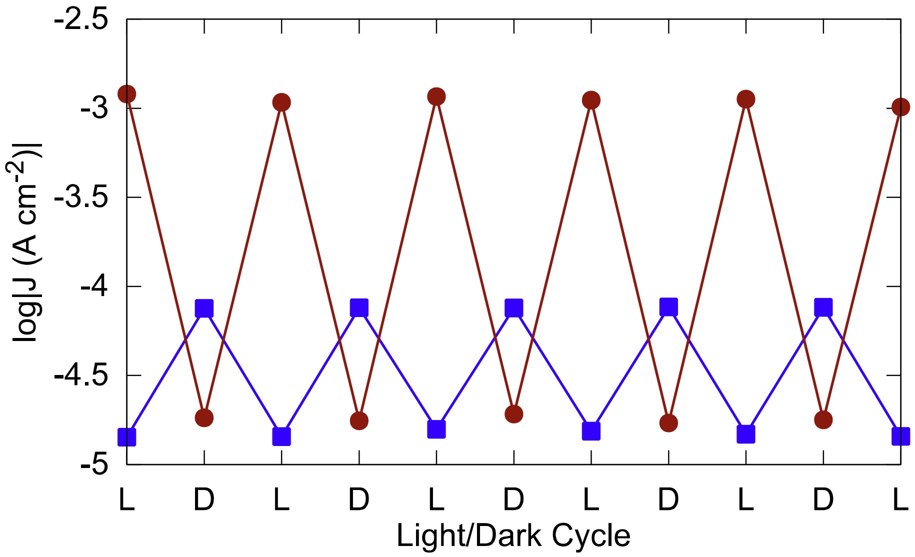

Supplementary Figure 4: Current density through AminoPyr and C18 nanojunctions during repeated light dark cycling. The values of log|*J*| at +0.5V as a function of light/dark cycles over a range of 10 switching cycles showing that AminoPyr (red circles) switching correlates to light but that C18 (blue squares) does not.

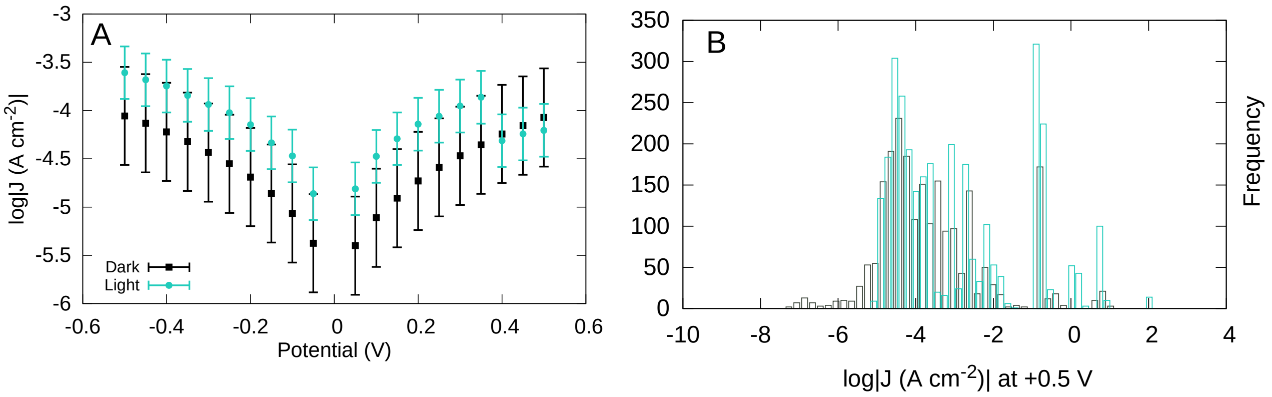

Supplementary Figure 5: Statics of current density voltage data. A: Current-density versus voltage plots of OMePyr-dark (black squares) and OMePyr-light (cyan circles). The points correspond to Gaussian means from fits of histograms of log|J| for each value of V. The error bars are the 95% confidence intervals computed from the standard deviation. B: Histograms of log|J| at +0.5 V in the light (cyan) and dark (grey) showing no change in the population. See supplementary note 1 for further description of junction fabrication and discussion of the results.

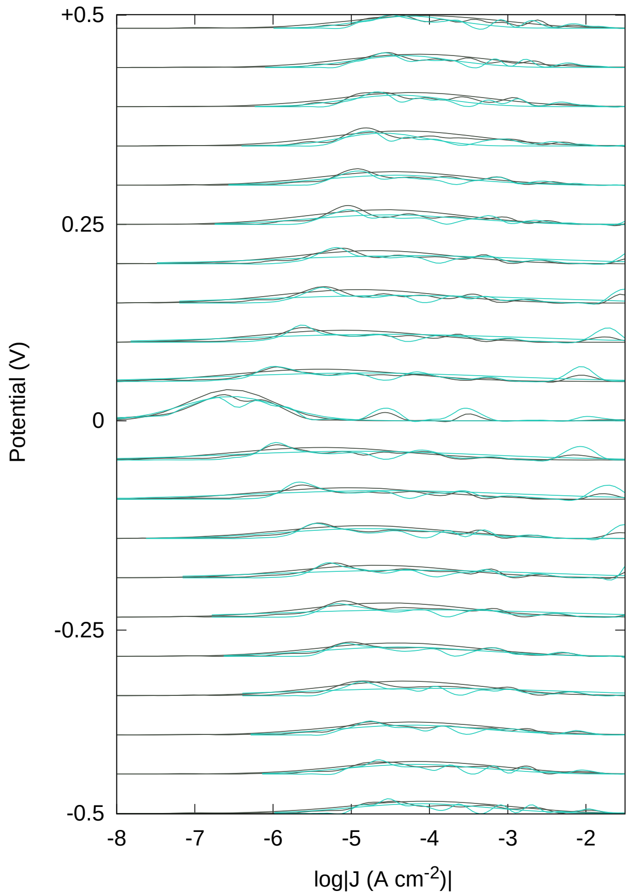

Supplementary Figure 6: Voltage–current characteristics of OMePyr nanojunctions. Pseudo-3D plots of the raw OMePyr data showing histograms (solid lines) and Gaussian fits (filled curves) of all of the data for OMePyr-dark (black) and OMePyr-light (cyan). The pseudo-Y axis corresponds to the potential for each histogram and the X-axis are the bins in units of log|J(A cm^{-2})|, *i.e.*, the plot resembles a rotated by 90 °.

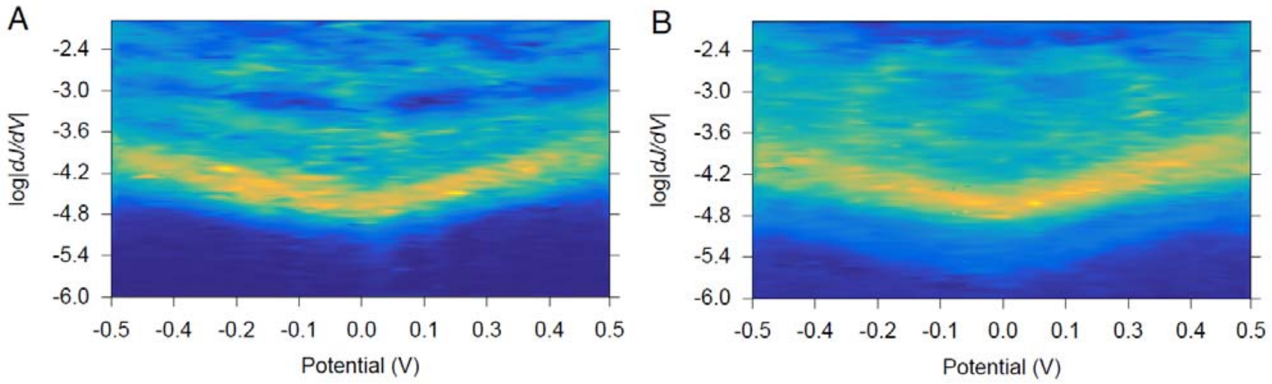

Supplementary Figure 7: Conductance "heatmap" plots for OMePyr. The plots showhistograms binned to log|dJ/dV| (conductance in Acm^{-2} V^{-1}, Y-axes) versus potential (in V, X-axes). The colors correspond to the frequencies of the histograms; lighter colors indicate higher frequencies. Panel A on the left shows OMePyr-dark and right panel B shows OMePyr-light on the right. These plots show two regimes of conductance, centered around –4.0 and –3.5, but the population inversion is not as pronounced as it is with AminoPyr.

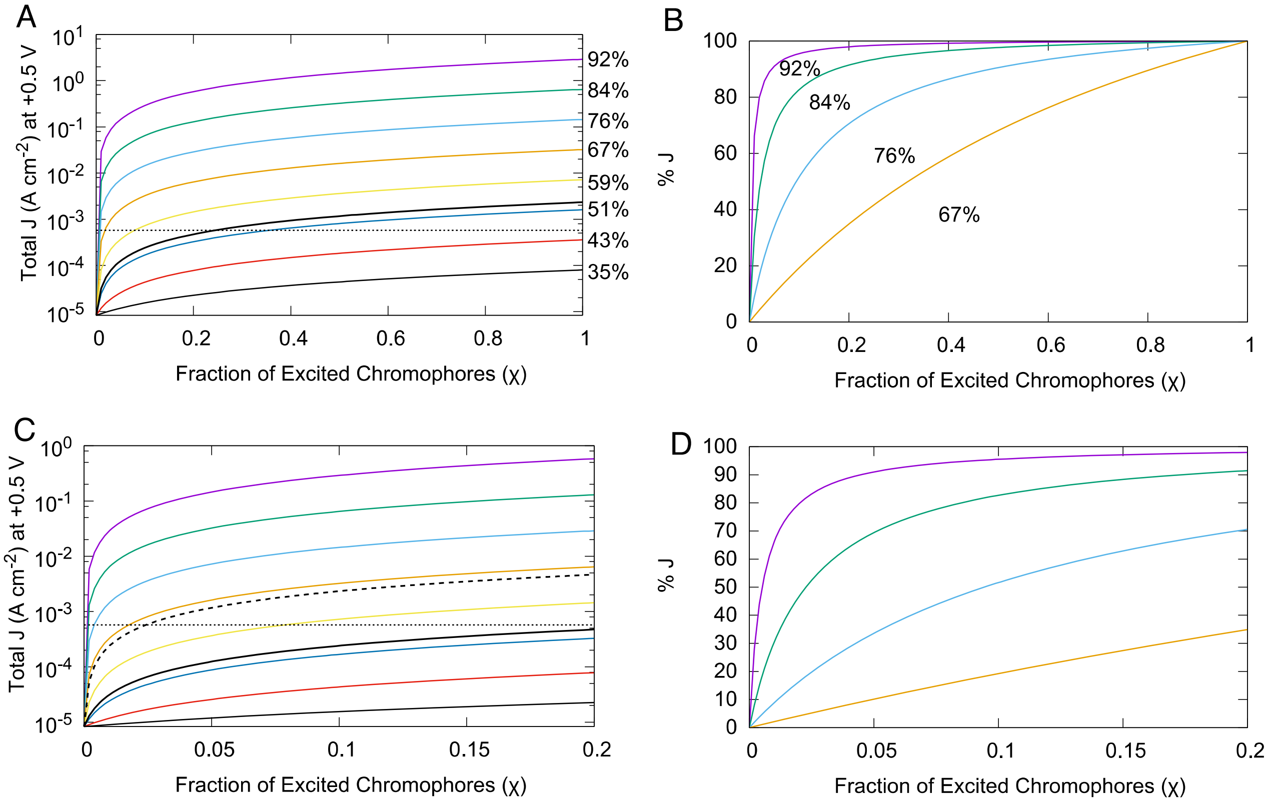

Supplementary Figure 8: Plots of Supplementary Eq.1 with J_0= 10^7 A cm^{-2}, β= 0.75 Å^{-1} and J_i= 10$^{-5.08}$ A cm^{-2} taken from Supplementary Refs 6 and 7. The percentages refer to the decrease in tunneling distance compared to the length of AminoPyr of 24.5 Å. The plots A and C on the left show the total current flowing through a junction with different effective tunneling distances. The horizontal dashed lines indicate the experimental value of J_i for AminoPyr-lightand the thick black lines correspond to the distance of the alkyl tail. The plots B and D on the right show the percentage of J that is carried by the fraction of molecules with smaller effective tunneling distances. The upper plots A and B show $0 \leq \chi \leq 1$ while the lower panels C and D cover $0 \leq \chi \leq 0.2$. The black, dashed line in C is the curve using Δd for AminoPyr and β= 0.55.

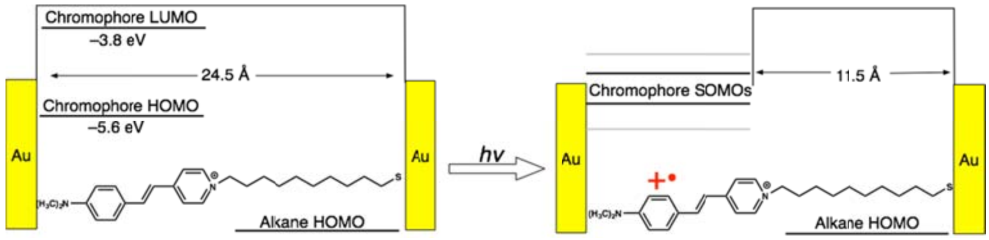

Supplementary Figure 9: **Effective tunneling distance**.The cartoon shows how the effective tunneling distance could drop from 24.5 to 11.5 Å if the absorption of light created a hole (or electron) on the chromophore of AminoPyr. This current would be too small to observe directly, but given a steady-state fraction of AminoPyr in the excited state of ≥0.05 it would lead to the observed increase in *J*. The small population of increased values of *J* observed in the dark would arise from thermal population of the excited state. The values for the HOMO and LUMO were taken from Supplementary ref.1. This mechanism is directly supported by the DFT calculations presented in the Main Text.

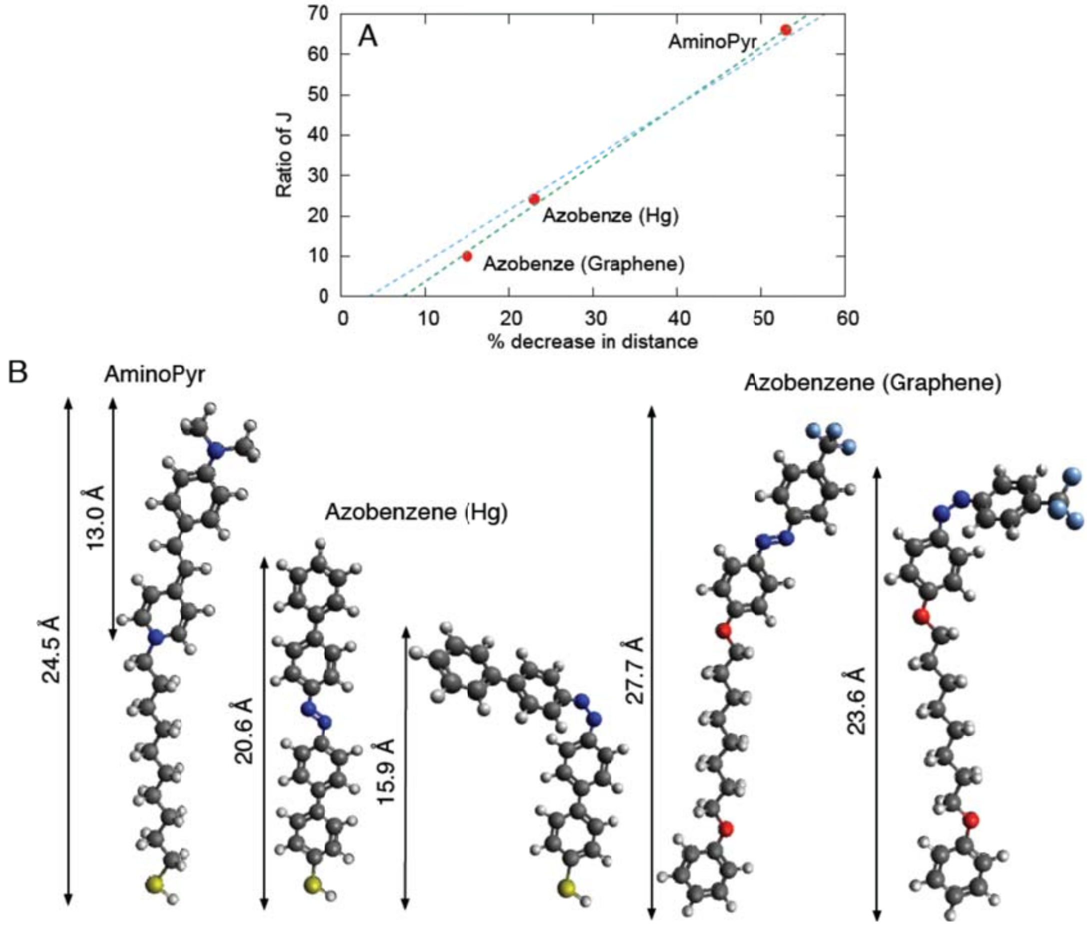

Supplementary Figure 10: Estimating the change in tunneling distance required to explain light induced changes in current density. A: plot of the ratio of *J* versus the corresponding change in *d* for this work, according to the model above, and for data from Supplementary references6 and 7 using Supplementary Eq. 1, setting $\chi = 1$. The dashed lines are linear fits of $y(x) = 1.45x - 10.72$, $R^2 = 1.0$ (experimental values) and $y(x) = 1.29x - 4.32$, $R^2 = 0.98$ (including a point at 0,0 for no switching). B:Structures from which the percentage decrease in lengths were estimated.

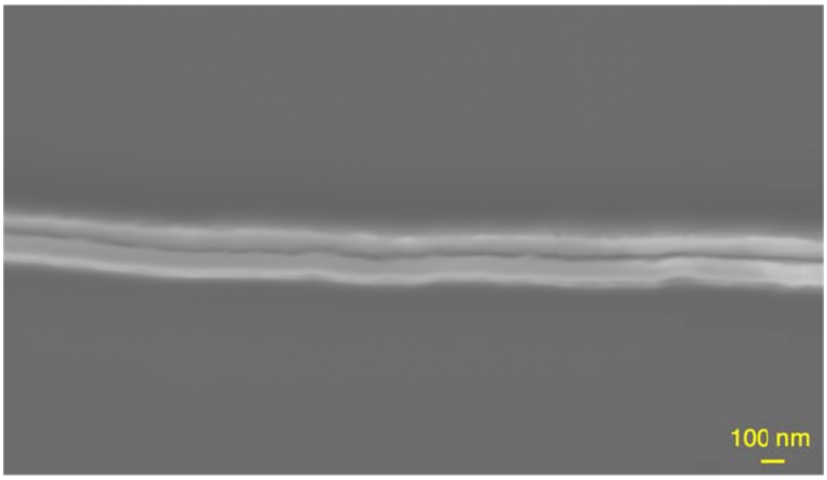

Supplementary Figure 11: SEM image of a STAN device containing AminoPyr showing part of the nano-gap between the electrodes. The gap extends for approximately 500 µm and the wires are 2-4 cm long making the devices impossible to visualize in their entirety. Extensive characterization of STANs is shown in Supplementary Refs 4 and 5. The apparent size of the gap is exaggerated by the angle (the actual width is below the resolution of the SEM and is therefore not resolvable edge-on).

Supplementary Note 1: Cycling of conductance.

Supplementary Figure 3 shows the values of log|*J*| plotted against 1000 light/dark cycles for AminoPyr and C18. These are the entire datasets used to generate all of the plots in the Main Text and Supplementary Information. While little quantitative interpretation is possible, qualitatively there is a clear difference in the two plots; AminoPyr shows significantly more events than C18. These plots also reinforce the importance of the statistically analysis presented in the Main Text for which we could have analyzed only a slice of the dataset that showed perfect switching for ~100 cycles.

Supplementary Figure 4 shows a section of Supplementary Figure 3 for which C18 is perfectly out of sync with the light/dark stimulus, while AminoPyr remains in sync. From the histograms of *J* for AminoPyr-dark it is clear that AminoPyr was also out of phase some of the time, but C18 switched randomly, while the gating effect for clearly statistically correlated to the presence/absence of illumination. One possible explanation is the, for C18, the dominant effect is the heading of the leads, which increases the series resistance (but not enough to counteract the increase in conductivity of AminoPyr-light).

Supplementary Note 2: Fabrication and characterization of OMePyr nanojunctions.

We initially set out to characterize the photo-gating phenomenon by measuring a full series of hemicyanine dyes that vary by counterion and donor/acceptor strength, however, the SAMs of all but two proved to be too fragile to survive the fabrication process (*i.e.*,they yielded only short circuits). Of those two, only AminoPyr was robust enough to interrogate rigorously. Despite the low yield of working devices, we were able to collect a sufficient amount of data on OMePyr to construct conductance histograms and, therefore, conductance heatmaps. This difference is small enough that the histograms in the light and dark overlap considerably, thus the population inversion is not readily apparent and, while the peaks of the histograms are distinguishable, we cannot say that they differ with statistical significance (Supplementary Figure 5). The cyanine dye OMePyr is

identical to AminoPyr except that $N(CH_3)_2$ is replaced by OCH_3; *i.e.*, it pairs a weaker donor with the same acceptor. This difference is clearly visible in the ~1 eV shift in the photon energy corresponding to maximum absorption in the visible range.[1] The magnitude of the change in conductance upon irradiation (at any wavelength) of OMePyr is about a factor of 5, as compared to 100 for AminoPyr. Although the histograms of J for OMePyr-dark and OMePyr-light are shifted (Supplementary Figure 2), the J/V plots in Supplementary Figure 5A show that the confidence intervals of $\mu_{\log}$overlap, meaning we cannot differentiate this apparent switching behavior from random chance. It does, however, stand to reason that the magnitude of the change in conductance would correlate to the relative strength of the donor/acceptor if the origin of the photo-gating is molecular. We can reasonably rule out electrode effects (*e.g.*,heating) because C18shows no signs of photo-gating, only random statistical fluctuations, and simply heating the leads would cause a lower conductivity in the light.

We include this analysis both because it is the only other chromophore-containing STAN electrode that did not give exclusively short-circuits and because it highlights the statistical significance of the gating effect in AminoPyr. Supplementary Figure 5 reveals how $\mu_{\log}$ for OMePyr could be misinterpreted; the apparent difference in magnitude is simply due to a band of low-conductance data at ~ –4.8 that is present in the dark, but not under illumination. This difference is a random statistical fluctuation that is clearly distinguishable from the effect in STANs of AminoPyr. Given the similarity in the magnitude of OMePyr-dark and AminoPyr-dark, we hypothesis that the STANs of OMePyr did not show a gating effect either because the magnitude of the charge-transfer absorption was too weak or because the SAMs were damaged non-catastrophically during the fabrication process.

Supplementary Note 3: Magnitude of Switching.

There are a many different models for tunneling transport through molecules attached to metallic leads. They fall into two general categories; those that model$J$ through a junction empirically and those that consider the detailed electronic structure of the molecule. The latter, as described above, usually considers the equilibrium (zero bias) conductance of a single molecule as a scattering site in close proximity to two electrodes through which current flows. These models rely on detailed quantum mechanical descriptions of the scattering site (*e.g.*, DFT calculations) and, although single-molecule calculations can give information about transmission features, it nearly impossible to apply these methods directly to SAMs, which comprise molecules in many different conformations induced by grain boundaries, electrode defects, impurities, etc. Thus, empirical models are generally used to describe, for example, trends in J across SAMs of systematically differing molecules.

A simple model for tunneling transport SAMs in which a fraction of the molecules in the SAM is less resistive to tunneling currents is to model the total current using the simplified Simmons equation, $J = J_0 e^{-\beta d}$ where d is the effective tunneling distance, β is the tunneling decay coefficient and J_0 is the theoretical value of at d=0. This is a deep tunneling model that assumes a rectangular tunneling barrier that summarizes the electronic detail of the SAM as β, which is a constant for a particular type of backbone (*e.g.*, CH_2). Therefore, to describe areas of the SAM that are more conductive, d is varied to account for a change in the effective tunneling distance, while leaving β constant. (The parameter d actually describes the width of the barrier though it is often assumed to describe the distance between electrodes, an assumption that is only valid for deep tunneling.) These areas are called "thin-area defects," but can be used to describe any region of a SAM that exhibits a smaller effective tunneling distance, *i.e.*, that is more conductive to tunneling current. Weiss et al.[2] modeled the total observed current density J_{total} using Supplementary Eq. 1 where χ is the fraction of thin-area defects (more conductive molecules) and J_i is the nominal current density

of the pristine SAM. Using a consensus value of J_0 from the literature,[3] a value of β determined for alkanes in STAN electrodes,[4] a value of J_i for AminoPyr-dark at 0.5 V and d as the length of AminoPyr, we plotted J_{total} and the percentage of J_{total} carried by thin-area defects in Supplementary Figure 4 by varying the effective tunneling distance, d, which is expressed as the percentage decrease compared to AminoPyr-dark (Δd). The total tunneling current is highly sensitive to the change in the effective tunneling distance; at Δd=67% and χ=0.2, J_{total} increases by ~ 10^2. At the same time, the contribution to J from the thin-area defects increases rapidly; at Δd=67% and χ=0.1, 30% of J will be carried by the more conductive molecules.

$$J_{total} = (\chi)J_0e^{-\beta d} + (1-\chi)J_i \quad \text{Supplementary Equation (1)}$$

To apply this simple model to STANs of AminoPyr, we consider AminoPyr-dark to correspond to the defect-free SAM of nominal conductance ($J=10^{-5.08}$ A cm^{-2} at 0.5 V) and the measured J to correspond to J_{total}. When light is shined on the STAN, the SAM between the electrodes rapidly reaches a steady-state in which the fraction of chromophores in the excited state (χ) is constant and J_{total} increases because these charged chromophores (AminoPyr-light) are more conductive than in the ground-state. Regardless of the dynamics (*i.e.*, the spatial distribution of excited chromophores is constantly changing), χ will remain constant and therefore the excited chromophores can be considered "thin-area defects." We do not need any detail of the mechanism of transport beyond the assumption that it is dominated by non-resonant tunneling (which we established experimentally [4,5] and which is indicated by the shape of the conductance heatmaps) and that, therefore, we can use a reasonable a value of β. Thus, to model the relative change in tunneling resistance between AminoPyr molecules in the low- and high-conductance states, we assign as lower effective tunneling distance to the latter.

The average observed values of J for AminoPyr-dark and AminoPyr-light at 0.5 V was $10^{-5.08}$ and $10^{-3.24}$ A cm^{-2} respectively. According to Supplementary Figure10, to achieve this increase would require a Δd of 59% at χ=0.5 or 76% at χ=0.01. Estimating reasonable values of χ is difficult without knowing the mechanism of energy transfer or the lifetime of relaxation, but if we assume that charges generated in the SAM are stabilized by image charges in the electrodes, χ=0.2 is high, but not unreasonable. (Screening is effective in SAMs because the decreased dimensionality causes dipolar interactions to become short-range.) At this value of $\chi\Delta d$ would need to be ~40-50%. The AM1-minimized end-to-end distance of AminoPyr is 24.5 Å, the hemicyanine chromophore is 13.0 Å and the alkyl tail is 11.5 Å. Since the alkyl portion does not interact with light, only the 53% of the total length of the molecule that is made up chromophore can be considered to alter J_{total} under irradiation. Thus, to account for a Δd of 54%, the chromophore would have to contribute almost no tunneling current in the excited state; *i.e.*, J_{total} would be dominated by the alkyl tail. This situation is not unreasonable if the chromophores in the excited state come into resonance with the electrode. For example, if the photo-excitation drives the injection of an electron into the electrode, although the photocurrent itself would be too small to measure, it would leave a hole behind on the chromophore, which would then function as an extension of the (Fermi energy of the) electrode not unlike Au/PEDOT:PSS or EGaIn/Ga_2O_3; *i.e.*,the SOMO of the chromophore would pin to the electrode and the width of the tunneling barrier would decrease to the length of the alkyl tail. A cartoon of this interpretation is shown in Supplementary Figure 9. The small population of increased values of J in the dark (*i.e.*, the electrical bi-stability) would correspond to the small fraction of thermally excited chromophores that always exists at equilibrium. Although this model is crude, it can explain the observed increase in J using reasonable numbers taken from experimental data. Note that β has an enormous impact on the plots in Supplementary Figure 8. We chose a value of 0.75 Å^{-1}, which was derived from STANs of alkanedithiols and is probably higher than the actual value for AminoPyr. Taking a value of 0.55 Å^{-1} lowers the critical value of χ to 0.025 (the value referenced in the Main Text). This curve is shown in the lower-left plot in Supplementary Figure 8.

To put our proposed model in context with literature data, we plotted the measured ratio of *J* against the percentage change in effective distance for AminoPyr together with data from Supplementary references 6 and 7 in Supplementary Figure 10A. The latter two papers report the conductance switching of azobenzene moieties–*i.e.*, switching via photoisomerization–in which the change in distance is taken as the difference in length between the *cis* and *trans* forms. These values are approximations based on the values of *J* taken from published graphs and are from disparate systems; a graphene/monolayer/graphene device and Au/SAM/Hg junctions. Nonetheless, there is an apparent linear relationship, which implies that the effective change in *d* for AminoPyr may correspond to a physical change in distance. The fit is not perfect because the Y-intercept (*b*) has to be zero (*J* can only change if *d* changes); the fit to the experimental values gives R^2=1.0 and *b*=–10.72. Fitting the same data with a point at 0,0 gives R^2=0.98 and *b*=–4.32, which is again surprisingly good for such a simple model.

Supplementary Note 4: DFT Calculations.

We simulated STAN electrodes comprising AminoPyr using two six-atom Au clusters as electrodes and by truncating the alkyl chain to two carbons. The "bottom" electrode is coupled through a S-Au bond at a FCC hollow site and the "top" electrode is physisorbed. The density of states and transmission curves were calculated with Gaussian 09 using the B3LYP/LANL2DZ in accordance with literature procedures.[8] To capture the transmission features in the excited state, we calculated the first triplet excited state and the radical cation that would result from quenching the photo-excited state by electron transfer. Ideally excited-state calculations are done using TD-DFT, but TD-DFT does not produce a Fock matrix for the excited states, thus transmission calculations would only reflect the ground state. We computed transmission for both spins and they did not differ. Although the density of states is identical for AminoPyrin the excited and cationic states, the SOMO energies differed greatly, which is reflected in the additional positive resonance near E_f. Although relaxation times will differ greatly depending on the local environment, vertical excitations and vibrational relaxation occur on the order of picoseconds. These calculation, therefore, support the hypothesis that the change in conductance occurs on the picosecond timescale because the tunneling probability is higher for AminoPyrin the excited and oxidized states.

Supplementary Methods

A technical-grade 3" silicon wafer was treated in an air plasma cleaner for 30 seconds and then was exposed to (tridecafluoro-1,1,2,2,-tetrahydrooctyl)trichlorosilane vapor for one hour. A layer of gold (100 nm-thick, which defines the width of the wires) through a Teflon master (that defines the length of the resulting wires; 1.5 mm) was deposited onto the pre-treated silicon wafer and this layer would serve as one of the final nanowires. The entire wafer was covered with ~8.5 mL of Epofix epoxy pre-polymer and was cured for three hours at 60 °C. The gold layer that is attached to the epoxy was template stripped. A 1 mM solution of hemicyanines, 1 equivalent of 1,8-diazabicyclo[5.4.0]undec-7-ene (DBU) as base and 1 equivalent of tri-n-butyl-phosphine was made in methanol. Putting the first layer of gold in this solution in a closed chamber that is purged with nitrogen overnight allows the creation of the self-assembled monolayer (SAM) on top of it. After taking out the sample out of solution, rinsing it with methanol and chloroform and placing it in the oven at 60 °C for a two minutes, the Teflon mask was placed back onto the epoxy substrate, but laterally offset by ~80% of the shortest dimension of the gold features. A second layer of gold was

deposited through the mask (100 nm-thick in this case). This layer of gold serves as the second gold nanowire. The Teflon mask was removed, taking care not to scratch the features, which will result in broken nanowires. Then the entire substrate was re-embeded in Epofix pre-polymer (~ 8.5 mL) and was cured for at least three hours at 60 °C. Cutting this cured material with saw results in the strips of the desired structures which were placed in the polyethylene mold (Electron Microscopy Sciences), were embeded in epoxy and were let to be cured so that the blocks for mounting on the ultramicrotome would be ready.

After trimming the block surface of the sample to the width of the diamond knife (we used 4 mm Diatome Ultra 35 °) with the razor blade in a trapezoid shape, it was sectioned with diamond knife which was mounted on ultramicrotome (Leica EM UC-6). The sectioning speed was adjusted at 1mm/s for the desired thickness of 100 nm. The sections of the gold layers embedded in epoxy float on the surface of the water in the boat of the knife. The epoxy sections containing the structures were collected from the surface of the water in the reservoir of the knife as ribbons of several sections to a substrate by placing substrate under the surface of the water and raising it slowly. After gathering the sections, they were put in the oven at 60c so that it would improve the adhesive of the sections to the substrate.

The substrate was placed under a light microscope or the stereoscope attached to the ultramicrotome and drops of silver paste (or carbon ink) were applied on two ends of wires in each section. These embedded metallic structures will be visible as either a black line (from the gold/epoxy interface) or, in the case of thicker gold structures (from the deposition steps), directly visible. In either case, the drops were applied sufficiently far from the center not to short the nano-gaps. Then, the substrate was placed in a home-build Faraday cage and one of the electrodes was grounded using a small drop of Ga-In to connect the pad of silver paste to a tungsten probe. The other electrode was contacted by positioning a syringe with a sharp tip of Ga-In over the other pad of silver paste, bringing it in contact, and connecting the syringe to the electrometer. (Both drops of Ga-In served as vibration isolation and soft contacts.) Electrical measurements were performed sequentially in the dark and under illumination. (Dark meaning under ambient lights, in a Faraday cage with the lid down.) *J/V* data were collected for each device in the mentioned sequence as follow: 1) Five scans in dark (no irradiation with lasers), 2) Five scans under Irradiation with green laser (λ= 532 nm), 3) Five scans in dark, 4) Five scans under irradiation with purple laser (λ= 405 nm), 5) Five scans in dark, 6) Five scans under irradiation with red laser (λ= 650 nm). This cycle was repeated 100 times or until the device shorted. We did observe the irradiation/no irradiation cycles for more than 100 cycles and the junctions are still stable and responsive. The STAN devices are more sensitive to fatigue from *J/V* sweeps than from light cycling.

Characterization. Supplementary Figure 1shows the UV-visible absorption spectra of AminoPyr in and in a SAM on Au. The three wavelengths of lasers used, 405, 532, and 650 nm are indicated on the plot (which is in eV). Both 405 and 650 nm fall outside of the absorption of AminoPyr in solution, but the spectrum broadens sufficiently on Au to place all three wavelengths in the absorption window. The absorption at 650 nm is considerably weaker than for the other two wavelengths. If the mechanism of excitation were the direct absorption of a photon by AminoPyr, one would expect a lower magnitude of switching at that wavelength (according to the model outlined below). If excitation occurs via energy transfer from absorption of the electrodes, however, the trend becomes much less straightforward to intuit. Extensive characterization of SAMs of AminoPyr is shown in Supplementary Ref.1.

Supplementary Figure 11is a scanning electron micrograph of a STAN device containing AminoPyr. The total length of the wires is 1-2 cm and the overlap that forms the gap is ~500 µm, which makes imaging them in their entirety impossible. Thus, Supplementary Figure 11only shows a section of the gap, but it is clear that it follows the contours of the electrodes. The apparent gap appears larger than it is because it is viewed at an angle to enhance the edges. Viewing face-on does

not resolve the gap sufficiently because it is below the resolution of the instrument. More extensive characterization of STANs including TEM data that prove the width of the gaps correponds to the thickness of the SAM/template can be found in Supplementary Refs 4 and 5.We use this methodology to determine that the gap is fully intact in the STANs of AminoPyr; the most straightforward of which is the fact that other SAMs in the hemicyanine series[1] that did not show any tunneling current. That is, from that series, only AminoPyrand OMePyrsurvived the fabrication process. In fact, even SAMs of AminoPyr with different counterions (*e.g.*, I^-) did not form STANs.

Statistics. A common method of error-analysis for large-area, SAM-based tunneling junctions is to construct histograms of J for each value of $V$and then to report the peak, $\mu_{\log}$, and the standard deviation. This method of analysis shows the value of $\mu_{\log}$ that was observed with the highest frequency and, through the experimental design, asserts that $\mu_{\log}$ is near to the real value of $\log|J|$ for that junction/SAM. Thus, it reports the accuracy of the measurement as $\mu_{\log}$ and the precision as $\sigma_{\log}$. This method of analysis is useful for comparing different SAMs or junctions, but does not capture the statistical difference between values of J measured for the same junction under different conditions, for which a statistical test against the null hypothesis that the difference in J is due to random chance is more appropriate. Thus, in addition to reporting the histograms and fits of each junction/SAM (Supplementary Figure 11) we computed the confidence intervals.

A confidence interval is the range over which the difference between the population parameter (that describes the Gaussian) and the observed value of $\mu_{\log}$ is not statistically significant at the 5% level (*i.e.*, a *t*-test at 0.05). It also means that, if a new set of devices were measured and the confidence interval calculated, there is a 95% chance that population parameter would fall inside that interval. Thus, the confidence interval does not predict the value of $\mu_{\log}$ for future measurements, but it does mean that the values of $\mu_{\log}$ in the dark and under irradiation for the observed devices and events differ statistically significantly at the 95% confidence level (which describes the reliability of the estimate and is not related to $\mu_{\log}$). The key difference between reporting $\mu_{\log}$ and $\sigma_{\log}$ for a single histogram and computing the confidence interval is that the latter expresses a range of possible values of $\mu_{\log}$ that cannot be ascribed to random chance, but nothing about the probability of finding a particular value of $\mu_{\log}$; the former is concerned with accuracy and precision, while the latter is concerned with probability.

$$CI = \mu_{log} \pm A\frac{\sigma_{log}}{\sqrt{n}} \quad \text{Supplementary Equation (2)}$$

The confidence intervalsCI for $\mu_{\log}$ depicted as error bars in the *J/V* plots were calculated using Supplementary Eq. 2where $\sigma_{\log}$ is taken from Gaussian fits, e.g., Supplementary Figure 2, n+1 is the number of devices measured and A is taken from a standard table of *t*-distributions (e.g., 2.2626 for n=9 at the 95% confidence level). This is a sensible analysis for photo-gating data because it expresses a range of possible values of $\mu_{\log}$ from a dataset; it tests whether or not two parameters ($\mu_{\log}$ in the light and dark) differ by random chance.

***J*/*V* Analysis**. Data were acquired as described above and then filtered by discarding short- and open-circuit traces. The data were then parsed in a "hands-off" manner using Scientific Python to produce histograms of J for each value of V, the associated Gaussian fits (using a least-squares fitting routine) and the conductance heatmap plots. These Gaussian fits are shown in Supplementary Figure 2. For the heatmap plots, $G = \log|\frac{dJ}{dV}|$ was computed from un-smoothed numerical derivatives from which histograms of G for each value of V were constructed. The data in the heatmap plots were interpolated from Gaussian fits to the histograms of G (using a least-squares fitting routine) to provide data for values between experimental values of V.

The *J/V* plot for AminoPyr could not be constructed in the same hands-off manner because of the presence of two, distinct distributions in the histograms of J. The data for C18 and OMePyr could be fit as a single Gaussian because only some of the values of V showed bimodal histograms (and

even then the difference was not nearly as clear-cut as for AminoPyr). Thus, for the *J/V* plot of AminoPyr the peak values are $\mu_{\log}$ of the main Gaussian from a two-peak fit and the confidence interval is calculated from $\sigma_{\log}$ of that Gaussian. All other calculations and plots (including Supplementary Figure 2) are taken directly from the output of the Scientific Python script.

Supplementary References

1) Vijayaraghavan, R. K., Gholamrezaie, F. & Meskers, S. C. J. Photovoltaic effect in self-assembled molecular monolayers on gold: Influence of orbital energy level alignment on short-circuit current generation. *J. Phys. Chem. C***117**, 16820-16829 (2013).

2) Weiss, E. A., Chiechi, R. C., Kaufman, G. K., Kriebel, J. K., Li, Z., Duati, M., Rampi, M. A. &Whitesides, G. M. Influence of defects on the electrical characteristics of mercury-drop junctions: self-assembled monolayers of n-alkanethiolates on rough and smooth silver. *J. Am. Chem. Soc.***129**, 4336-4349 (2007).

3) Simeone, F. C., Yoon, H. J., Thuo, M. M., Barber, J. R., Smith, B. &Whitesides, G. M. Defining the value of injection current and effective electrical contact area for EGaIn-based molecular tunneling junctions. *J. Am. Chem. Soc.* **135**, 18131-18144 (2013).

4) Pourhossein, P. & Chiechi, R. C. Directly addressable sub-3 nm gold nanogaps fabricated by nanoskiving using self-assembled monolayers as templates. *ACS Nano***6**, 5566-5573 (2012).

5) Pourhossein, P. & Chiechi, R. C. Fabricating nanogaps by nanoskiving. *J Vis. Exp.* e50406 (2013).

6) Seo, S., Min, M., Lee, S. M. & Lee, H. Photo-switchable molecular monolayer anchored between highly transparent and flexible graphene electrodes. *Nature Comm.* **4**, 1920 (2013).

7) Ferri, V., Elbing, M., Pace, G., Dickey, M. D., Zharnikov, M., Samorì, P., Mayor, M. &Rampi, M. A. Light-powered electrical switch based on cargo-lifting azobenzene monolayers. *Angew. Chem. Int. Ed.* **47**, 3407-3409 (2008).

8) Herrmann, C., Solomon, G. C., Subotnik, J. E., Mujica, V. & Ratner, M. A. Ghost transmission: How large basis sets can make electron transport calculations worse. *J. Chem. Phys.***132**, 024103 **(2010).**
